# Functional Complementation of *sir2Δ* Yeast Mutation by the Human Orthologous Gene *SIRT1*

**DOI:** 10.1371/journal.pone.0083114

**Published:** 2013-12-11

**Authors:** Davide Gaglio, Anna D’Alfonso, Giorgio Camilloni

**Affiliations:** 1 Department of Biology and Biotechnology Charles Darwin, Sapienza-Università di Roma, Rome, Italy; 2 Istituto Pasteur - Fondazione Cenci Bolognetti, Sapienza-Università di Roma, Rome, Italy; 3 Institute of Molecular Biology and Pathology, The National Research Council (CNR), Rome, Italy; National Taiwan University, Taiwan

## Abstract

Sirtuins, class III histone deacetylases, are proteins homologous to the yeast protein Sir2p. Mammalian Sirt1 has been shown to be involved in energy metabolism, brain functions, inflammation and aging through its deacetylase activity, acting on both histone and non-histone substrates. In order to verify whether Sirt1 can replace Sir2p in the yeast cells, we expressed the full-length human Sirt1 protein in *S.cerevisiae sir2Δ* mutant strain. The structure of chromatin is basically maintained from yeast to human. Thus, yeast chromatin is a favourable environment to evaluate, inhibit or activate an ectopic histone deacetylase activity in an *in vivo* substrate. Mutant *sir2Δ* shows a series of different phenotypes, all dependent on the deacetylase activity of Sir2p. We analyzed the three silent loci where normally Sir2p acts: ribosomal DNA, telomeres and the mating type loci. Moreover, we verified extrachromosomal ribosomal DNA circles production and histone hyperacetylation levels, typical marks of *sir2Δ* strains. By strong *SIRT1* overexpression in *sir2Δ* cells, we found that specific molecular phenotypes of the mutant revert almost to a wild-type condition. In particular, transcriptional silencing at rDNA was restored, extrachromosomal rDNA circles formation was repressed and histone acetylation at H3K9 and H4K16 decreased. The complementation at the other studied loci: HM loci, telomere and sub-telomere does not occur. Overall, our observations indicate that: i) *SIRT1* gene is able to complement different molecular phenotypes of the *sir2Δ* mutant at rDNA ii) the *in vivo* screening of Sirt1 activity is possible in yeast.

## Introduction

Sirtuins, class III histone deacetylases (HDAC III), are proteins homologous to the yeast protein Sir2p. Enzymes belonging to this family show strong NAD-dependent activity and are involved in the control of a series of basic functional and metabolic pathways. These activities have been reported for almost all organisms, from bacterial to human, in which they have been found [1,2].

In particular mammalian *SIRT1*, the S. *cerevisiae* orthologue of *SIR2*, has been shown to be involved in important pathways, including energy metabolism, brain functions, inflammation and aging [1,3].

In humans, seven sirtuin family members (*SIRT1-7*) have been described, with different roles and cellular localizations. As far as the enzymatic activity of sirtuins is concerned, Sirt1, Sirt2, Sirt3, Sirt5 and Sirt6 deacetylate proteins at lysine residues [2,4,5], while Sirt4, Sirt6 and Sirt7 are the only sirtuins that show a strong ADP-ribosylation reaction [2,5].

There is recent evidence that Sirt7also exhibits deacetylase activity [6]. Deacetylation activity requires the metabolic cofactor NAD^+^, and the final products of the reaction are the deacetylated protein, nicotinamide (NAM) and O-acetyl-ADP-ribose [7]. The metabolic cofactor NAD^+^, which sirtuins activity rely on, directly connects the cellular energetic status with the chromatin structure and with the transcriptional repression [3]. For this reason sirtuins are considered a putative enzymatic system that may adapt genetic programs to the metabolic status of the cell [2,3].

Sirt1 is the best-characterized member of the family, itacts on a wide variety of protein substrates, including histones and shows a high percent identity with the yeast protein Sir2p [2]. 

Moreover, human Sirt1 and yeast Sir2p have a partially overlapping specificity for histone residues, specifically for H4K16Ac and H3K9Ac [2].

However, Sirt1 also deacetylates transcription factors and cofactors, triggering their activation or inactivation, with relevant consequences on gene expression [8]. In mammals, Sirt1 activity has been associated with a series of disease-related processes, e.g., chromatin/epigenetic modifications in neural functions (Parkinson’s and Alzheimer’s diseases), metabolism (Diabetes syndrome), cancer (prostate cancer), cardiovascular function and inflammation or stress responses [1,2,3]. 

The relevance of the sirtuin family is supported by the ever-growing number of studies in the literature. In addition, the discovery of a series of molecules that inhibit or activate sirtuins has had a strong impact on biological and biomedical research [8,9].

Excellent reviews describing both the biology and the chemistry of sirtuins are available [1,11,12].

When considering the wide implication of sirtuins in biomedical research, the possibility to obtain specific and potent regulators becomes an important pursuit that will certainly open up new therapeutic perspectives. In spite of the fact that inhibitor and activator molecules have been found very early in the history of sirtuins, *in vivo* assays on chromatin substrates are still missing. At present, Sirt1 activity assays are based on in vitro deacetylation reaction with peptide substrates [13]. We decided to express the *SIRT1* gene in *S. cerevisiae* since the yeast model presents many advantages [14,15], i.e. the availability of specific mutants, the highly characterized genetic environment and the easy genetic manipulation. In view of these observations we deemed this simple biological model capable of furnishing detailed information on the basic mechanisms of Sirt1 enzymatic reaction in vivo. 

A previous study by Sherman J.M. et al. [16] described the molecular cloning of a human sirtuin in *S. cerevisiae sir2Δ* mutant. Specifically they did not express Sirt1 but hSir2Ap, also known today as Sirt2 [16,17]. They discovered that only a chimera with N/C-terminal of yeast Sir2p and the core portion of hSir2A (human Sirt2) was able to replace Sir2p activity on a subset of genetic loci. Actually, Sirt1 shows a higher percent identity with yeast Sir2p than Sirt2 and is considered the phylogenetic ortholog of yeast Sir2p [2]. Although Sirt2 still shows a high similarity with the yeast protein Sir2p, it is considered more similar to another yeast sirtuin, Hst2p [2].

In this study we expressed the full-length form of *SIRT1* in yeast *sir2Δ* mutant cells under the inducible GAL1 promoter, and studied the complementation of *sir2Δ* molecular phenotypes.

The yeast *sir2Δ* mutant shows a series of different phenotypes, all depending on the deacetylase activity of Sir2p. The phenotypes we considered were: transcriptional silencing at *HMLα1* gene, the telomeric transcript *YFR057W*, the subtelomeric* IRC7* lying on TEL VI, and the ribosomal non coding transcripts of both *NTS1* and *NTS2* regions. We also studied other phenotypic marks of the *sir2Δ* strain such as extrachromosomal rDNA circles (ERCs) production and histone hyperacetylation levels. 

Here we showed that ectopic *SIRT1* expression is able to rescue some of *sir2Δ* mutation phenotypes with different efficiency in different chromosomal regions.

Our study demonstrated that human Sirt1 in *S. cerevisiae* acts on some chromatin substrates, and therefore, the yeast system may be exploited for screening

## Results

### *SIRT1* cloning, expression and toxicity in *S. cerevisiae*

**Figure 1:**
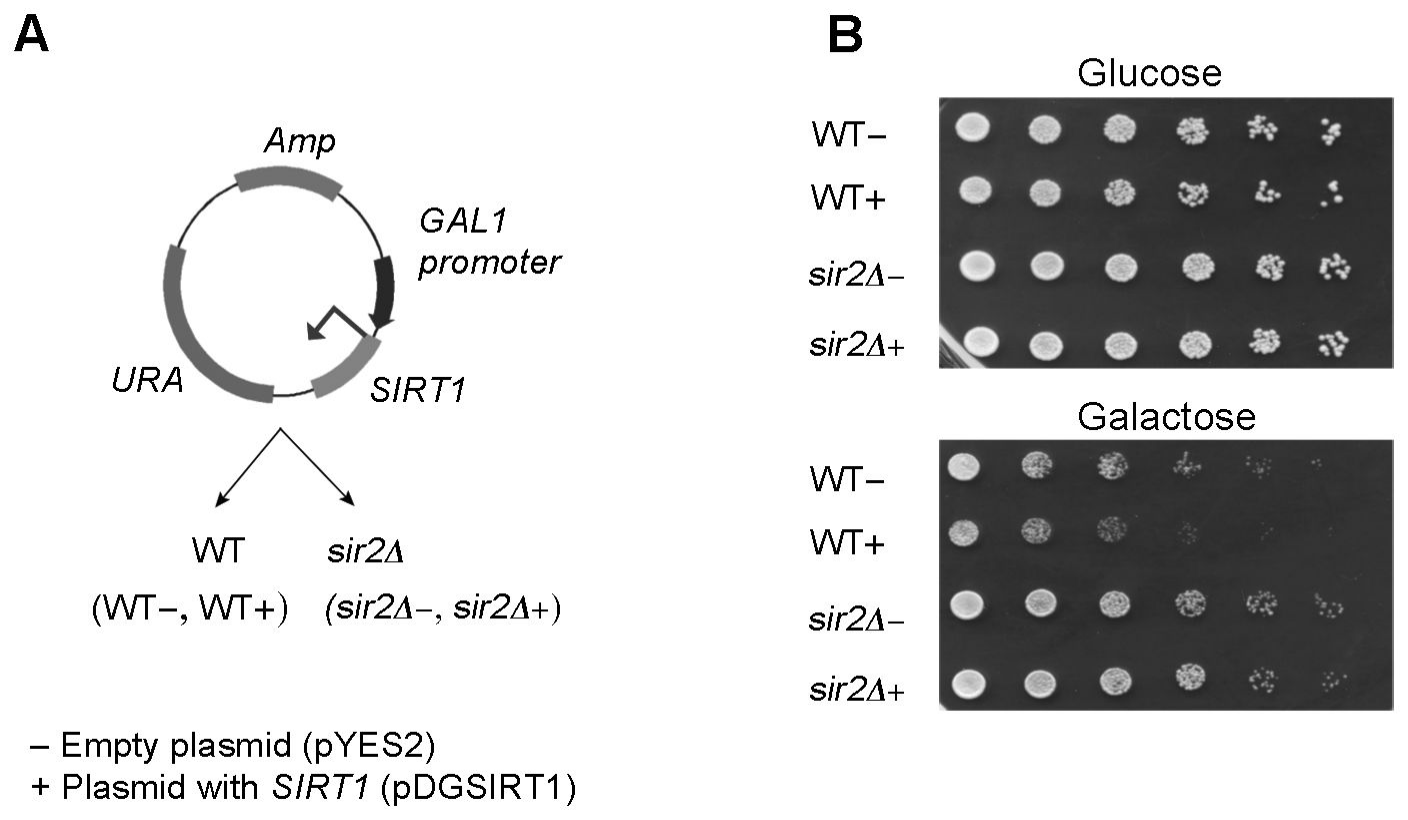
SIRT1 construct and its toxicity in yeast cells. (A) Construct for yeast expression with *SIRT1* under the inducible promoter GAL1 in pYES2 background. (+: SIRT1 construct; −: empty plasmid). (B) Yeast spot test analysis of growth phenotypes during plasmid repression and induction conditions (glucose and galactose, respectively). For each strain five-fold serial dilutions were made and 5 μl were spotted onto minimal medium plates.

In order to verify whether the full length human *SIRT1* gene complements all or part of *sir2Δ* mutant phenotypes in *S. cerevisiae*, we inserted the *SIRT1* coding sequence from p1791 plasmid [18] into the pYES2 plasmid to yield pDGSIRT1 (Figure 1A; details in M&M). WT and *sir2Δ* cells were transformed with pDGSIRT1 (+), or with the empty vector (-) and a dilution spot assay was performed (Figure 1B). Yeast cells grown to logarithmic phase were initially diluted to 4×10^3^ cells/μl. Six serial five-fold dilutions were made and 5 μl of each were spotted onto minimal medium plates containing glucose or galactose as carbon source, and then incubated at 30°C. Both strains transformed with empty or *SIRT1* gene-containing vectors did not show significant differences in growth efficiency when plated on glucose-supplemented medium. However, when galactose plates were analyzed, a reduction in colony forming efficiency was evident in WT+ cells when compared to WT-, (Figure 1B).

We also cloned the non-catalytic version of Sirt1, (Sirt1-H363Y), into the pYES2 plasmid to check which complemented phenotypes are due to the catalytic activity of the human protein (Figure S1, D). The *SIRT1-H363Y* containing plasmid, pDGSIRT1-H363Y, has been used to transform *sir2Δ* cells in order to yeld the control strain *sir2Δ*+*. We further tested the toxicity of this non-catalytic Sirt1 version by spot assay (Figure S1, E). The *sir*2Δ- (empty vector), *sir2*Δ+ (catalytic Sirt1) and *sir2*Δ+* (non-catalytic Sirt1 version) strains did not exhibit growth defects when plated on glucose medium or in galactose medium (Figure S1, E).

Taken together, these data suggest that Sirt1 and the non-catalytic mutant Sirt1-H363Y are not toxic in *sir2Δ* mutant cells. In WT+ cells, however, a slight decrease in cell growth was observed when compared to WT-, possibly attributable to the physical or genetic interaction between the endogenous Sir2p and the ectopically expressed Sirt1, (Figure 1B).

**Figure 2:**
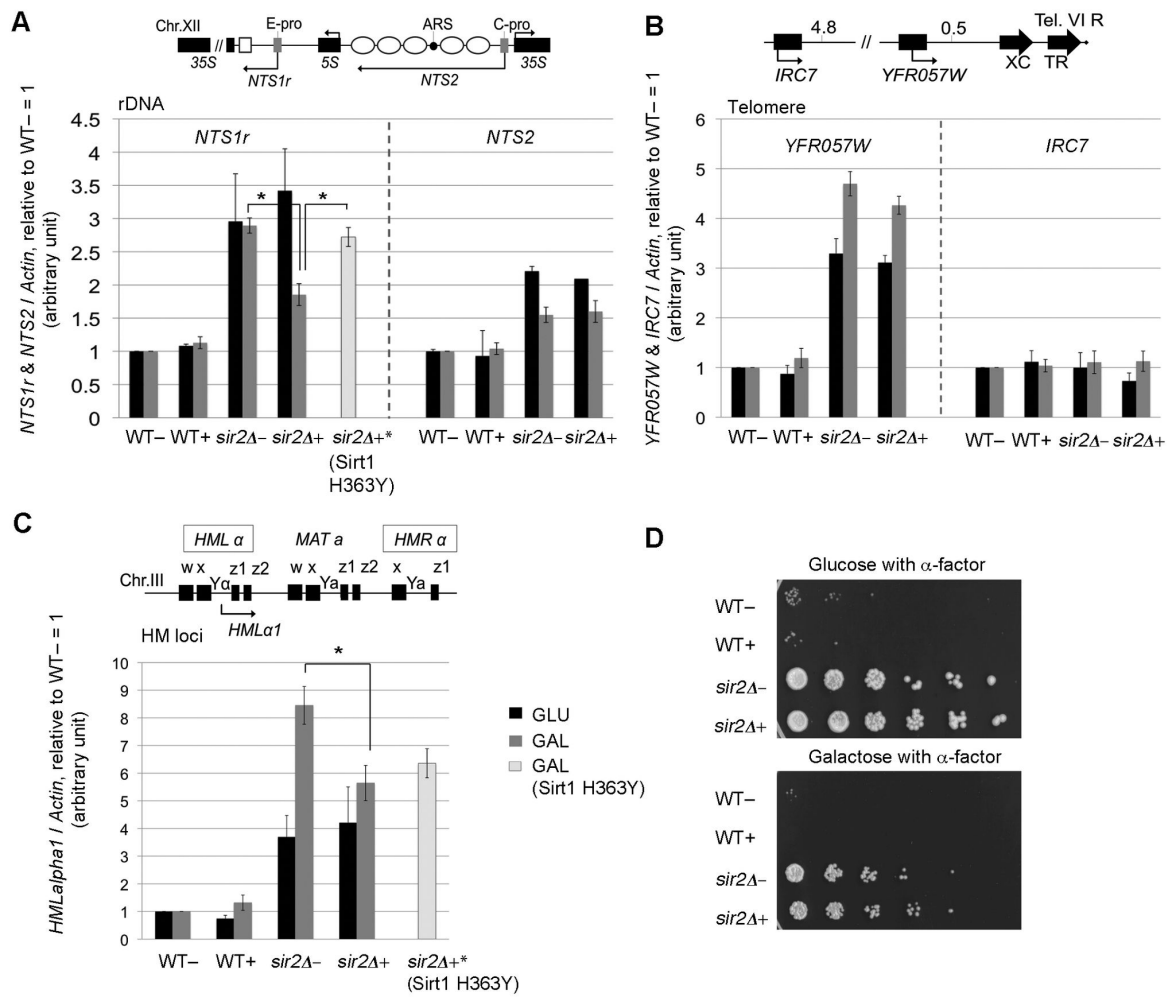
Global histone acetylation is influenced by Sirt1. (A) Western blot analysis of H3K9, H4K16, H4K12 global acetylation after *SIRT1* overnight induction. *SIRT1*, H3Ct, H4Ct and α*TUB* hybridization were performed as induction or loading controls. (B) Western blot quantification of panel A. Histograms indicate averages and Std. Dev. bars from 4 biological replicates (H3K9Ac **p < 1%, H4K16Ac p = 10.3%, H4K12Ac p = 27.2%). (C) Western blot analysis of H3K9Ac on the same strains that in panels A and B but also with the strain *sir2Δ*+* containing the non catalytic mutant *SIRT1*-*H363Y* as control. (D) Western blot quantification of panel C. Histograms indicate averages and Std. Dev. bars from 4 biological replicates, (H3K9Ac, *sir2Δ*- *versus sir2Δ*+, ** p<1%; *sir2Δ*+* *versus sir2Δ*+, ** p<1%; Sirt1/Sirt1-H363Y, *sir2Δ*- *versus sir2Δ*+ or *sir2Δ*+ *, ** p<1%; Sirt1/Sirt1-H363Y, *sir2Δ*+* *versus sir2Δ*+, p>>5%). Statistical analysis as in Figure 3.

In order to verify the correct mRNA expression of *SIRT1* and *SIRT1-H363Y*, we analyzed their transcripts by reverse transcription PCR (Figure S1A). Moreover the presence of Sirt1 protein in strains transformed with empty plasmid (-), *SIRT1* gene-containing vectors (+) or *SIRT1-H363Y* gene (+*) was evaluated by Western blot. In figure S1, panels B-C show a time-course analysis of Sirt1 expression after switching the carbon source from glucose (GLU) to galactose (GAL) in the growth medium. Sirt1 expression was evident between 6 and 9 hours of GAL induction both in *sir2*Δ+ and *sir2*Δ+* cells. However, after overnight induction the protein was still abundantly present (Figure 2A). In order to analyze complementation phenotypes we also checked the protein expression level of the human Sirt1 and the non-catalytic version Sirt1-H363Y (Figure 2C, 2D). Figure 2 show that the different forms of the human protein in *sir2*Δ+ and *sir2*Δ+* cells are expressed at the same level (Figure 2D).

### *sir2Δ* complementation: ncRNA transcriptional silencing

*sir2Δ* mutation in *S. cerevisiae* is characterized by a series of typical phenotypes: i) loss of transcriptional silencing at the rDNA locus, telomeres and HM loci [19,20,21].; ii) hyper-recombination at rDNA locus [22]; iii) histone hyperacetylation at silenced loci [23]. We intended to verify whether all or some of these phenotypes are complemented by the introduction and overexpression of *SIRT1* gene into the yeast cells. 

**Figure 3:**
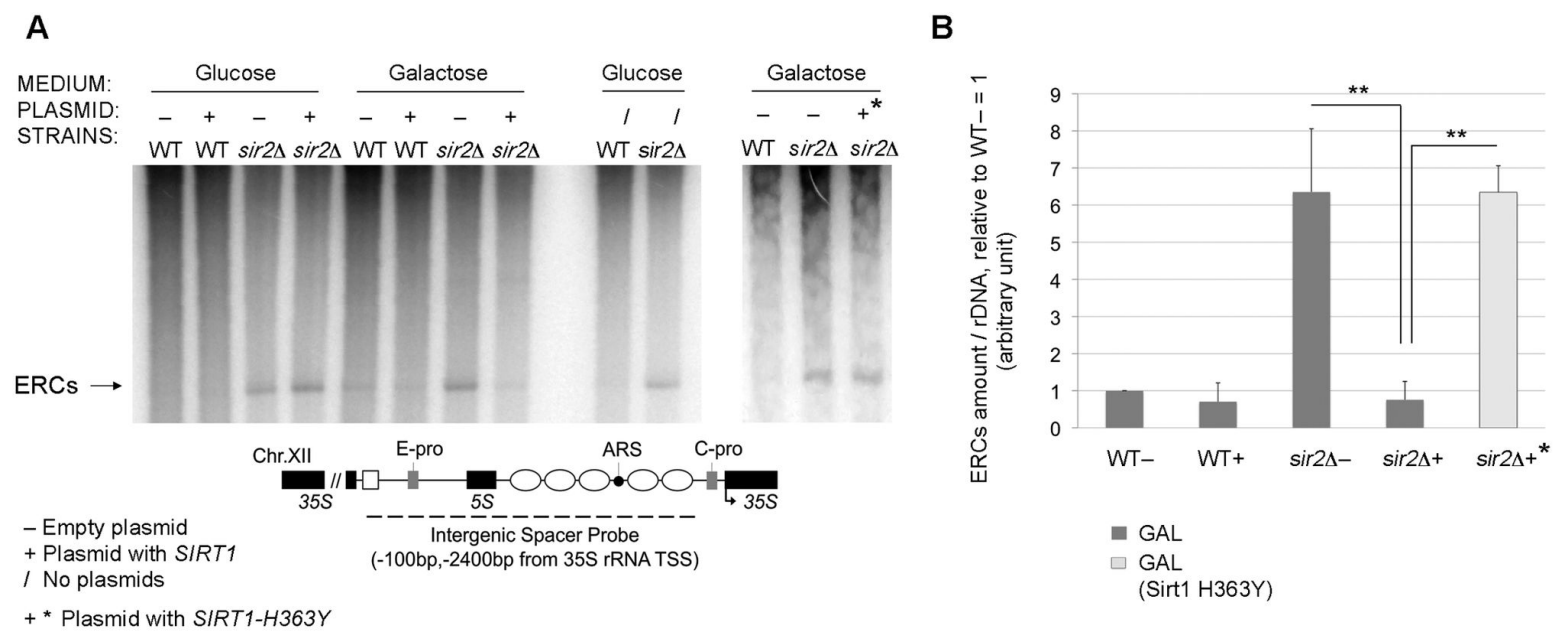
SIRT1 overexpression partially restores the transcriptional repression within specific loci in sir2Δ mutant. RT-PCR transcriptional analysis in WT and *sir2Δ* strains transformed with empty plasmid (-), *SIRT1* construct (+) or *SIRT1*-*H363Y* mutant construct (+*) both in repression (glucose) and induction (galactose) conditions. (A) rDNA locus: *NTS1r* and *NTS2*; (B) TEL VI locus: *YFR057W* and *IRC7*; (C) HM loci: *HMLalpha1*. Histograms indicate averages and Std. Dev. bars from at least three independent biological replicates. Two−tailed t−test was applied for statistical analysis. Asterisks indicate statistically significant differences between *sir2Δ*- and *sir2Δ*+ or between *sir2Δ*+* and *sir2Δ*+ in galactose medium; α = 0.05. (Percentages of p−value: *p< 5%, **p < 1%, ***p < 0.01%). (D) Alpha factor yeast spot test analysis to evaluate *HMLalpha1* silencing. Five-fold serial dilutions of mutant *sir2Δ* and WT cells transformed with *SIRT1*(+) or empty plasmid (−) were spotted onto minimal medium plates containing alpha-factor.

In order to assess whether the loss of transcriptional silencing in the *sir2Δ* mutant is rescued when *SIRT1* is expressed, we analyzed the expression profiles of different genes known to be silenced in a Sir2p-dependent manner. RNA was extracted from WT and *sir2Δ* cells transformed with pDGSIRT1 (+) or empty plasmid pYES2 (-), converted into cDNA and analyzed by PCR. Cells were grown in both galactose (*SIRT1* induction) or glucose (*SIRT1* repression) medium. Two transcripts from the NTS (Non Transcribed Spacer) region of the rDNA were studied: *NTS1r* and *NTS2*. These transcripts are synthesized by RNA polymerase II, starting from E-PRO and C-PRO promoters, respectively (map in Figure 3A), [24,25,26]. In WT cells the repression of both transcripts is maintained regardless of the galactose-induction of *SIRT1* gene. Conversely, in *sir2Δ* cells, where *NTS1r* and *NTS2* are expressed, Sirt1 induction partially silences *NTS1r* transcription (Figure 3A). In galactose, (gray histograms), the repression is efficient at the E-PRO promoter, while at C-PRO the expression level remains the same; either cells are transformed with empty or *SIRT1*-containing constructs (*NTS1r*, *sir2Δ+ versus sir2Δ*-, * p<5 %). As control we analyzed *NTS1r* transcription level in *sir2Δ*+* strain that expresses the non-catalytic form of Sirt1. Figure 3A shows that in *sir2Δ*+* strain, *NTS1r* transcription is maintained as in *sir2*Δ- strain, this experiment demonstrates that *NTS1r* RNA repression is strictly dependent on the catalytic activity of Sirt1 (*NTS1r*, sir2Δ+* versus sir2Δ+, * p<5 %). Moreover the silencing of *NTS1r* does not occur in *sir2Δ+* cells grown in glucose because the plasmid with *SIRT1* is repressed (Figure 3A, black histograms).

The same cDNAs were analyzed for telomeric silencing, by studying *IRC7* (sub-telomeric) and *YFR057W* (telomeric) genes (Figure 3, panel B for map details). The *IRC7* gene, not efficiently silenced by Sir2p [27], maintained its expression level in all the analyzed conditions. Conversely, the *YFR057W* gene in sir2Δ- cells showed loss of transcriptional silencing which is not rescued by *SIRT1* overexpression in galactose medium (Figure 3B, gray histograms), (*YFR057W*in galactose, *sir2Δ+* versus *sir2Δ*-, p>5 %).

We then analyzed the expression of the *HMLα1* gene (Figure 3C). We chose *HMLα1* transcript as indicator of HM loci derepression. Upon loss of silencing in *MATa* strain, both *HMLα1* and *HMLα2* transcripts are subsequently repressed by *α1* /a1 heterodimer but it has been demonstrated that *HMLα1* transcript remains still detectable [28]. Matecic et al. demonstrated that *HMLα1* expression is an optimal quantitative measure of HM loci expression in *sir2Δ* strain [28].

As reported for telomeric and ribosomal silenced genes, in WT cells *HMLα1* expression did not change after the *SIRT1* expression. In *sir2Δ* cells, however, we observed a mild decrease in *HMLα1* expression when *SIRT1* was induced. In galactose, the reduction between *sir2Δ*+ and *sir2Δ*- reaches a statistically significant level (*HMLα1*, *sir2Δ+ versus sir2Δ*-, * p<5 %). However, at *HMLα1* locus there is not a silencing effect during Sirt1 expression but only a slight decrease of transcription not comparable to a wild-type repression state (Figure 3C).

We then analyzed *HMLα1* transcription in sir2Δ+* control strain. Figure 3C shows that *HMLα1* transcription decrease observed in *sir2Δ+* is not reverted in *sir2Δ*+* strain (*HMLα1*, sir2Δ+* versus sir2Δ+, p>5 %). It is conceivable to hypothesize that the protein overexpression may have an indirect effect on the transcription of this locus without an involvement of the enzymatic activity of Sirt1.

Another experiment that proves that at *HMLα1* there is not an effective repression is the α-factor assay, a powerful test highly sensitive to the degree of chromatin silencing [28].

Alpha-factor is a pheromone that blocks cell growth when *HMLα1* is repressed [29]. Thus growth is allowed only when transcriptional silencing on *HMLα1* gene is lost. In figure 3D, effective growth is shown for all *sir2Δ* strain, regardless of *SIRT1* galactose-induced expression. This indicates that the slight transcriptional decrease of *HMLα1* in *sir2Δ+* strain, shown by RT-PCR (Figure 3C), is not enough to produce a non-growing phenotype. 

Altogether, the data reported in Figure 3 indicate that the human Sirt1 protein rescues silencing phenotypes in *sir2Δ* cells at the ribosomal locus (Figure 3A), while the HM loci and the telomeric regions do not present any complementation effect dependent on the catalytic activity of Sirt1 (Figure 3B-C). On the contrary we demonstrated that the effect on the rDNA locus, specifically on the *NTS1r* locus, is strictly connected with the catalytic activity of Sirt1 (Figure 3A). However, the silencing efficiency obtained by *SIRT1* overexpression did not reach WT- levels in any the studied loci.

### *sir2Δ* complementation: Reduction of ERCs formation

Together with the lack of transcriptional silencing at silent loci like HM, telomeres and rDNA, *sir2Δ* mutant shows hyperproduction of ERCs. The formation of extrachromosomal rDNA circles has been associated with increased recombination activity among ribosomal units, and considered a marker of replicative aging in *S. cerevisiae* [30,31].

In order to evaluate whether rDNA recombination, leading to ERCs formation, is reduced upon *SIRT1* expression in *sir2Δ* cells, we compared WT and *sir2Δ* cells transformed with the pDGSIRT1 (+) or the empty plasmid (−) as in the previous section. As further control, we also analyzed the *sir2Δ*+* strain that expresses the Sirt1-H363Y non-catalytic mutant. 

**Figure 4:**
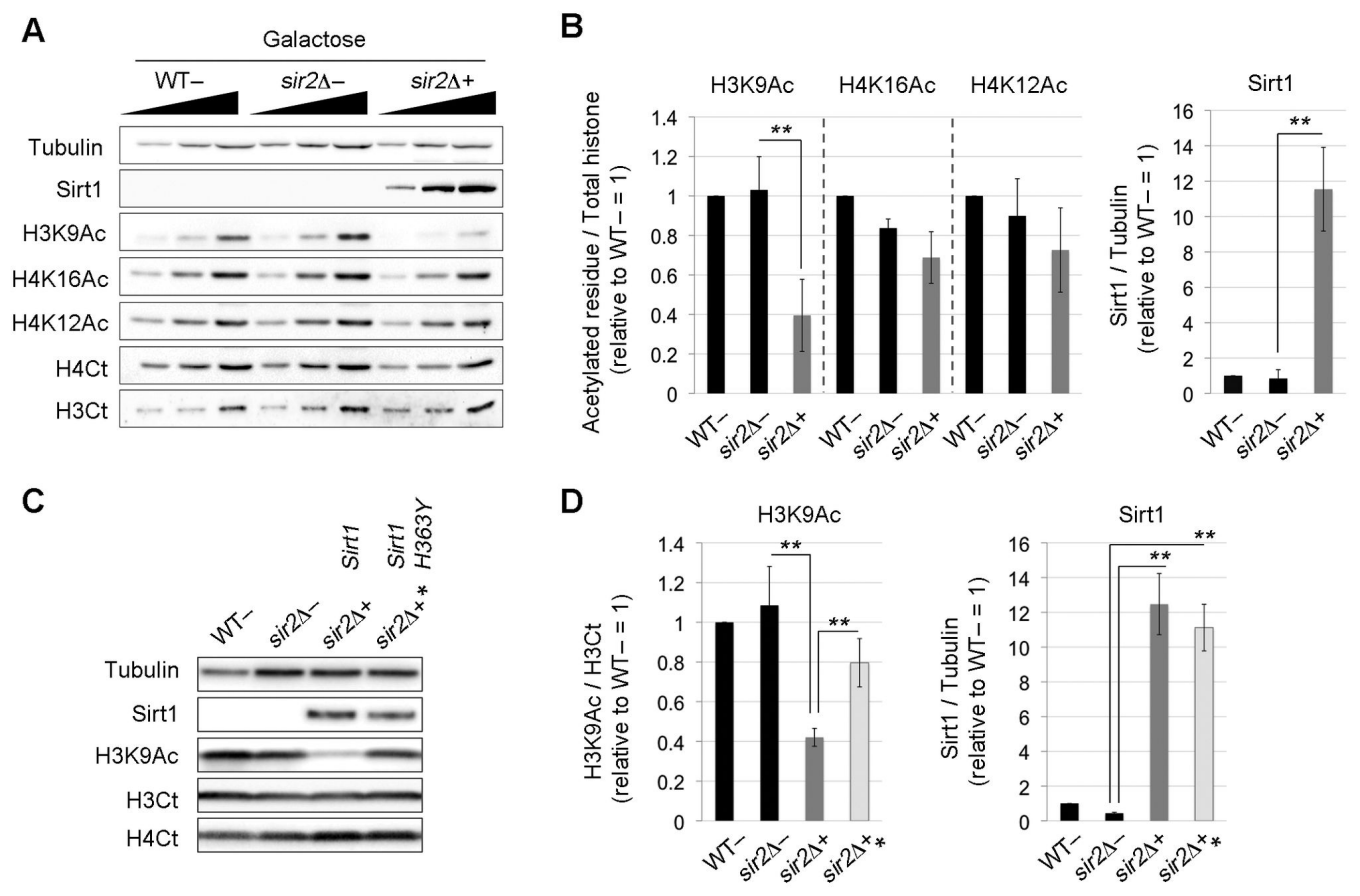
ERCs level decreases in complemented strain. A) Southern blot analysis of ERCs species in WT and *sir2Δ* with *SIRT1* construct (+), *SIRT1*-*H363Y* mutant construct (+*), empty plasmid (−) or without plasmid (/) as control. Strains were grown both in *SIRT1* repression and induction conditions (glucose or galactose, respectively). DNA was isolated from the specified yeast strains and probed with a radiolabeled rDNA sequence shown in panel (A). ERCs are indicated by an arrow. B) Quantification of ERCs amount in galactose condition: band intensities corresponding to ERCs were normalized to the hybridized bulk rDNA and referred to WT- levels. Histograms indicate averages and Std. Dev. bars from at least 4 biological replicates . Statistical analysis as in Figure 3.

DNA was extracted from cells in exponential growth phase (0.5 OD/ml) and subjected to agarose gel electrophoresis. After Southern blotting, the resulting nylon filter was hybridized to anrDNA intergenic spacer probe (map in Figure 4A), and visualized by autoradiography. In figure 4A, all WT(−,+) samples show a low amount of ERCs, while in *sir2Δ* samples the band corresponding to the ERCs species is evident. However, only in the *sir2Δ* sample transformed with pDGSIRT1(+) and grown in galactose (*SIRT1* overexpression), the amount of ERCs was reduced to WT- level. In addition this phenotype is reverted when we checked ERCs level in *sir2Δ*+* control strain. This control demonstrates that, as for *NTS1r* complementation phenotype, also ERCs repression is dependent on Sirt1 catalytic activity (Figure 4A).

Figure 4B reports the quantification of the ERCs species in galactose. (ERCs in galactose medium, sir2Δ+ versus sir2Δ-, ** p<1 %; sir2Δ+* versus sir2Δ+, ** p<1 %).

Data shown in Figure 4 demonstrate that the overexpression of human *SIRT1* in *S.cerevisiae* reduces ERCs production in *sir2Δ* strain and that this reduction is dependent on its catalytic activity.

### H3 lysine 9 acetylation is influenced by SIRT1

In order to analyze the *in vivo* role of Sirt1 in yeast cells, we performed a global acetylation analysis by quantitative immunoblotting. In these experiments we measured the effect of human *SIRT1* overexpression on the acetylation level of specific histone residues. In particular, we studied H3K9, H4K16 and H4K12, all common targets of the deacetylase activity of both yeast Sir2p and human Sirt1 [32,33,34,35].

Although yeast Sir2p has locus-specific roles such as the transcriptional silencing of rDNA, telomeres and HM loci [5,15,36], it is conceivable that Sirt1 has an effect at a global level, especially since the outcome of gene complementation between phylogenetically distant organisms is always unexpected. 

In yeast, Sir2p interacts with different protein partners according to the locus to be repressed [15,37,38]. The common mechanism for all targeted loci is the deacetylation of histone tails [23,39]. In fact, evidence shows that *sir2Δ* mutant is not characterized by high global acetylation levels, indicating that the Sir2p dominant role is played at specific loci [2]. In contrast, the mutation of another yeast-conserved sirtuin, Hst2p, displays high global acetylation levels probably affecting important processes such as the control of the cell cycle [2]. Since histones are highly conserved proteins and nucleosome structure is basically maintained unchanged from yeast to human we expected Sirt1 capable to deacetylate histones as well as Sir2p.

Cells from WT and *sir2Δ* transformed with the empty plasmid (−), and the *sir2Δ* strain complemented with the *SIRT1* construct (+) were grown in galactose overnight and analyzed by immunoblotting. In Figure 2, panels A and B show that after *SIRT1* induction in *sir2Δ+* strain, there is a strong decrease in H3K9Ac, (*sir2Δ+* versus *sir2Δ*-, **p<1%), whereas H4K16Ac and H4K12Ac do not show significant changes (H4K16Ac, H4K12Ac, *sir2Δ+* versus *sir2Δ*-, p=10.3% and p=27.2% respectively). The sir2Δ- strain transformed with the empty plasmid showed the same acetylation levels of the WT- strain. This experiment confirms that yeast Sir2p does not alter global acetylation levels, but rather acts in a locus-specific way. In particular, Sirt1 expression has an effect on H3K9Ac whereas it has no effect on H4K16Ac and H4K12Ac (Figure 2B). We also analyzed Sirt1 protein levels after overnight induction in galactose as an expression control (*sir2Δ+* versus *sir2Δ*- or WT-, ** p<1%) (Figure 2B). No Sirt1 signal was found in WT- or *sir2Δ−*. All histone acetylation quantifications were normalized to each specific total histone levels (H3K9Ac / H3Ct, H4K16Ac and H4K12Ac / H4Ct) and reported to the WT− strain = 1.

We used then the Sirt1 non-catalytic mutant to assess whether the strong H3K9Ac reduction was caused by the protein overexpression or by the Sirt1 enzymatic activity. We analyzed H3K9 acetylation in WT-, *sir2Δ*-, *sir2Δ*+ and *sir2Δ*+*, all grown in galactose medium. In figure 2, panel C and D show that H3K9Ac strong decrease, observed in *sir2Δ*+, is reverted in *sir2Δ*+* strain (H3K9Ac, *sir2Δ*- *versus sir2Δ*+, ** p<1%; *sir2Δ*+* *versus sir2Δ*+, ** p<1%). Moreover H3K9Ac decrease is properly caused by the enzymatic activity and not by the different levels of Sirt1 in the analyzed strains since *sir2Δ*+ and *sir2Δ*+* present the same expression level of the human protein (Sirt1/Sirt1-H363Y, *sir2Δ*+* *versussir2Δ*+, p>>5%),(Figure 2D).

### Histone deacetylation by Sirt1 at specific loci

**Figure 5:**
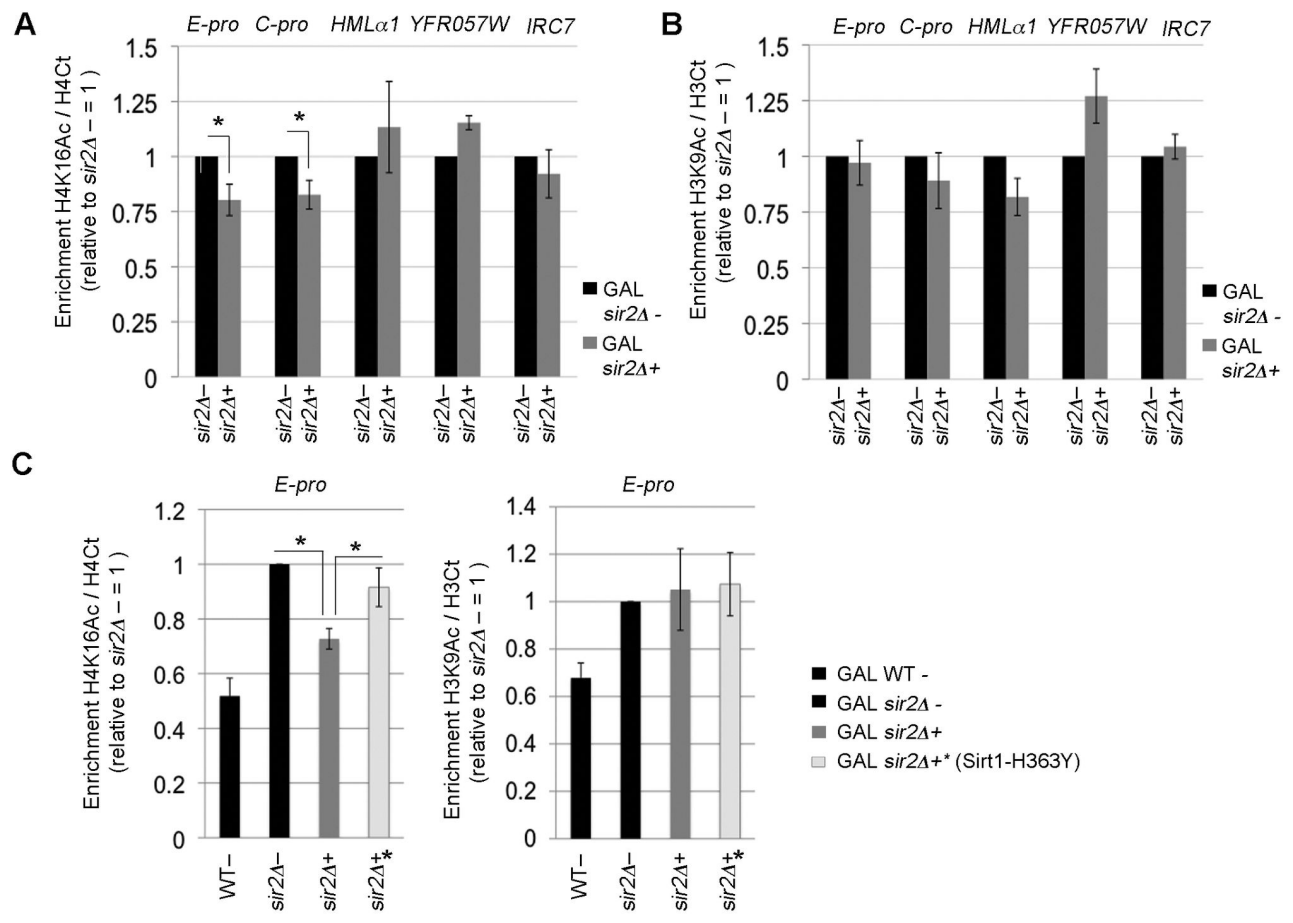
Sirt1 deacetylates specific loci. ChIp analysis of H4K16Ac (A) and H3K9Ac (B) at rDNA, HM loci, telomeric and sub telomeric regions in *sir2Δ*- and *sir2Δ*+ strains during *SIRT1* induction (galactose medium). (C) ChIp analysis of H4K16Ac and H3K9Ac at E-PRO in WT-, *sir2Δ*-, *sir2Δ*+ and *sir2Δ*+*. Acetylation enrichment for H4K16 and H3K9 were normalized to H4 C-terminal and H3 C-terminal, respectively, and referred to *sir2Δ*- levels =1. Histograms averages and Std. Dev. bars are representative of three technical replicates for at least three biological replicates performed. Statistical analysis as in Figure 3.

Since we demonstrated that Sirt1 expression reduces the global acetylation of H3K9 we further analyzed the acetylation of this residue by chromatin IP (Figure 5) within the three silenced yeast loci previously analyzed for specific RNA production (Figure 3).

Cells grown in galactose to exponential phase were treated with formaldehyde, then processed for ChIP analysis using antibody against the acetylated form of H3K9 and H4K16 or the C-terminal region of histones H3 and H4. The analysis was performed on WT-, *sir2Δ-, sir2Δ+* and sir2Δ+* strains, all grown in galactose medium to ensure the correct expression of *SIRT1.*

The immunoprecipitated DNA was amplified by PCR using specific oligonucleotides for the following regions: i) E-PRO and C-PRO (cryptic promoters of transcripts *NTS1r* and *NTS2, *respectively, within rDNA); ii) the coding sequence of the *HMLα1* transcript; iii) the *YFR057W* telomeric gene, highly repressed by Sir2p; and iv) the subtelomeric region *IRC7*, which is normally not silenced by Sir2p [27]. Since *sir2Δ*- mutants alter nucleosome occupancy within rDNA [26], we normalized H4K16Ac and H3K9Ac signals to those of H4 C-terminal and to H3 C-terminal respectively. All the data have been also normalized to the *sir2Δ−* strain = 1 (Figure 5). 

This analysis revealed that Sirt1 expression reduced acetylation of H4K16 in *sir2Δ+*, to a significant degree only in specific regions. The graph in Figure 5 shows a significant decrease both on E-PRO (*sir2Δ+* versus *sir2Δ*-, *p<5%) and the cryptic promoter C-PRO (*sir2Δ+* versus *sir2Δ*-, *p<5%). However, the acetylation of H4K16 does not decrease in *sir2Δ+*strain in HM loci as well as in *YFR057W* telomericand subtelomeric *IRC7* genes. 

As for the H3K9 acetylation, the statistical analysis did not reveal any significant changes in the studied loci (H3K9Ac, *sir2Δ+* versus *sir2Δ*-, *p>5%), (Figure 5B). 

It is interesting to observe that H3K9Ac and H4K16Ac may have different profiles in the same region; for instance, in rDNA cryptic promoters a general reduction of H4K16Ac prevails, while on the same region H3K9Ac does not exhibit any variation.

In view of these observations we used the *sir2*Δ+* strain to check if the acetylation decrease at the E-PRO promoter was dependent on the Sirt1 enzymatic activity. Figure 5C shows that *sir2*Δ+* strain, on the E-PRO region, exhibits a reversion of the *sir2*Δ+ phenotype for H4K16Ac but not for H3K9Ac, (H4K16Ac, *sir2Δ+* versus *sir2Δ*-, *p<5%; sir2Δ+* versus *sir2Δ+*, *p<5%)

**Figure 6:**
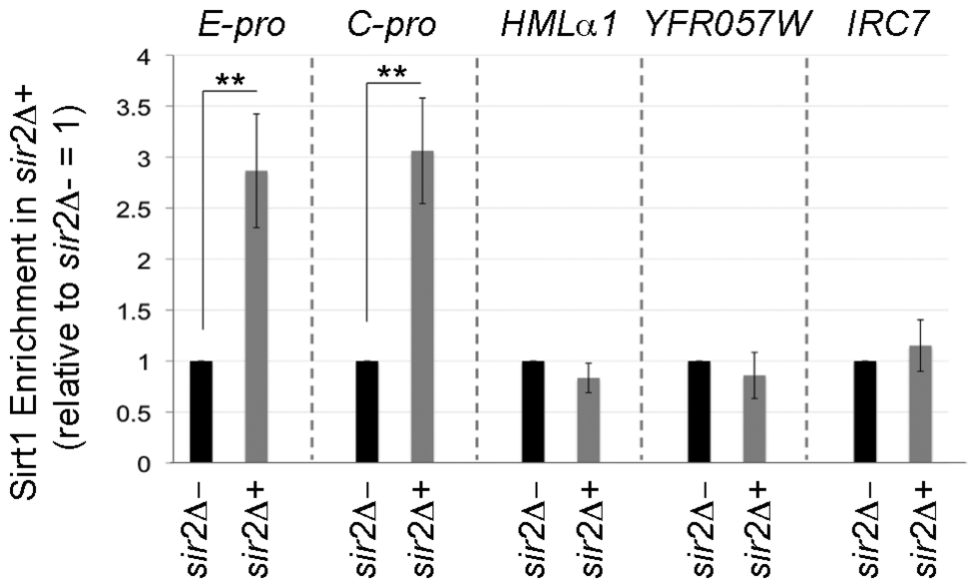
Sirt1 enrichment in specific loci. ChIp analysis of Sirt1 enrichment at rDNA, HM loci, telomeric and sub telomeric regions in *sir2Δ*+ and *sir2Δ*- strains during *SIRT1* induction (galactose medium). Sirt1 enrichment for *sir2Δ*+ was referred to *sir2Δ*- levels =1. Histograms averages and Std. Dev. bars are representative of three technical replicates for at least three biological replicates performed. Statistical analysis as in Figure 3.

We then analyzed also Sirt1 occupancy in the different studied regions (Figure 6). In order to maintain the correct expression of Sirt1, we harvested yeast cells in galactose as for the ChIP analysis of acetylated residues and we used *sir2Δ*- as negative control. Figure 6 shows how Sirt1 is more enriched in rDNA locus than the other analyzed regions. In particular, we detected a high enrichment on E-PRO and C-PRO in the rDNA locus both compared to *HMLα1, YFR057W*, *IRC7* in *sir2Δ*+ and to E-PRO in *sir2Δ*-, negative control (Figure 6), (Sirt1 enrichment, *sir2Δ*+ E-PRO versus *sir2Δ*- E-PRO, ** p<1 %). Here on E-PRO we previously observed three specific phenotypes: silencing of NTS1r (Figure 3A), reduction of ERCs formation (Figure 4) and acetylation decrease in H4K16 (Figure 5A). This experiment shows that Sirt1 is more enriched in this locus where the effective complementation occurs.

Taken together, these data further indicate that Sirt1 is able to act on the *S.cerevisiae* chromatin environment. However, four main points should be underlined: i) histone acetylation decreases at ribosomal locus; ii) At this locus Sirt1 is more enriched compared with the other regions iii) Sirt1 histone deacetylation differs from that of Sir2p in a region-dependent manner; vi) the locus where H4K16 acetylation is decreased, the rDNA, is the only region in which silencing is restored almost to a wild type level.

## Discussion

Sir2p from *S. cerevisiae* and human Sirt1 share a consistent amino acid identity [2]. Given the prominent role that Sirt1 plays as a master regulator of basic pathways both in healthy and pathological conditions [1,2,3], it would be extremely important to find further chemical regulators of this protein. However, to date, *in vivo* screening assays evaluating Sirt1 histone deacetylase activity are still missing. In view of these considerations, the complementation procedures developed here using *S*. *cerevisiae* as a model system could represent a new strategy in the search of chemical regulators of Sirt1. 

In this work, we studied the capability of human *SIRT1* gene to complement the mutation of the ortholog *SIR2* in *Saccharomyces cerevisiae*. In the literature, *sir2Δ* complementation experiments have been described using the human sirtuin Sirt2 that has lower percent identity with yeast Sir2p than Sirt1 [2,16,17]. In this study we used Sirt1, considered the phylogenetic ortholog of yeast Sir2p and we expressed it under the strong GAL1 inducible promoter to obtain clear-cut results. We also further analyzed the three silent loci where Sir2p normally acts: rDNA, Telomeres and HM loci; we employed techniques for the direct study of RNA silencing (RNA expression profiles were studied), DNA recombination (ERCs production was measured) and the extent of acetylation on specific target regions. This work revealed that *SIRT1* complements some of the *sir2Δ* molecular phenotypes at rDNA locus, while there was no recovery at other loci (HM loci and telomeric regions). We also demonstrated that this phenotype depends on the catalytic activity of Sirt1 (Figure 3).

Specifically, through transcriptional analysis, we demonstrated that during *SIRT1* induction the *NTS1r* is partially repressed whereas *HMLα1*, although presents a mild reduction is still highly transcribed if compared to wild-type strain (Figure 3A and 3C). Furthermore, we tested, by the transcriptional analysis of the non-catalytic mutant Sirt1-H363Y, if the complementation phenotypes we observed were caused by the enzymatic activity of Sirt1. This analysis revealed that the *NTS1r* decrease was attributable to the Sirt1 catalytic activity while as for HML*α1* it seems caused by an indirect effect of the overexpression. In fact in sir2Δ+* strain *NTS1r* transcription is restored as in *sir2Δ*- while HML*α1* is comparable to *sir2Δ+* (Figure 3A and 3C)*.*

In addition the alpha factor growth assay revealed that the *sir2Δ+* strain did not arrest its growth compared to the wild-type condition, even if the transcriptional analysis showed a slight reduction of the *HMLα1* transcript when compared to the *sir2Δ*- mutant (Figure 3C and 3D). A conceivable hypothesis is that the slight reduction of the *HMLα1* transcript is not sufficient to significantly reduce the expression of the protein and thus inhibit downstream pathways involved in alpha-factor responsiveness, even though the α-factor assay is a highly sensitive assay. As far as the telomere *YFR057W* gene and sub-telomere *IRC7* are concerned, despite the overexpression condition employed, Sirt1 protein seemed to have no transcriptional effect on these regions.

In addition this study shows, together with the decrease in *NTS1r*, a significant reduction of the production of extrachromosomal rDNA circles. In particular, by Southern blot ERCs analysis, we observed a complete reversion of the *sir2Δ* mutant phenotype (Figure 4A and 4B). Although the relationship between ERCs production, rDNA intergenic spacer transcription (*NTS1r* and *NTS2*) and its hyperacetylation has not been elucidated yet, they seem to be closely correlated [40,41]. 

Our data suggest that Sirt1 triggers the downregulation of the *NTS1r* intergenictranscriptbydeacetylatinghistones within the rDNA region (Figure 5). We hypothesize that as a consequence, the replication efficiency is reduced as well as the collision events between the replication fork and the transcriptional apparatus, which would lead to a reduced ERCs production (Figure 4). In the literature, the mechanism underlying the relationship between replication activity and ERCs formation has been clearly elucidated [42]. In addition, recent evidence indicates that several mutants show increased amounts of ERCs coupled with increased ncRNA production at rDNA[40].

Our study, by the use of full *SIRT1* in *sir2Δ* complementation, has also shown that the human protein can act on histone residues in a wide scale and not only in a locus-specific manner (Figure 2).

Specifically by Western blot analysis, we determined that Sirt1 is able to significantly reduce the global acetylation of H3K9. Since histones are the most conserved proteins in eukaryotic organisms, the existence of targets shared by both Sirt1 and Sir2p was rather expected and the global effect on histone acetylation in *sir2Δ-SIRT1* complementation was quite conceivable. 

We also demonstrated, by the use of the control strain sir2Δ+*, that the strong reduction of H3K9Ac is caused by Sirt1 catalytic activity indicating that this human protein is able to act on the yeast chromatin (Figure 2C).

Importantly this work deciphers the mechanism by which *SIRT1* complements the *SIR2* mutation in yeast through the modification of histone acetylation. We observed a decreased acetylation of H4K16 residue at rDNA locus (Figure 5) whereas HM loci, telomeric and subtelomeric genes were unaffected by Sirt1 overexpression, in fact these regions did not show altered H4K16 or H3K9 acetylation or mRNAs transcription. Interestingly, SIRT1 has a major impact on the global acetylation of H3K9 whereas in the complemented locus (the rDNA) it has effect mainly on H4K16Ac. 

The differences in H4K16Ac and H3K9Ac profiles we observed in the studied regions may depend on different kinds of *in vivo* interactions between Sirt1and the yeast proteins. In *S*. *cerevisiae* at HM loci and telomeres, Sir2p interacts with Sir3p, Sir4p and Rap1p, while at rDNASir2p is part of the RENT complex together with Net1p and Cdc14p [43,44,45]. Thus the acetylation pattern within the analyzed regions may depend on how the endogenous partner of Sir2p makes contact with the human protein Sirt1.

To verify Sirt1 enrichment in the different loci analyzed, we performed a chromatin immunoprecipitation experiment upon Sirt1-plasmid induction (Figure 6). This analysis revealed that Sirt1 has a different occupancy on different chromosomal regions studied. In particular the stronger enrichment is present at rDNA locus, here we have demonstrated the strongest complementation phenotype: the ERCs decrease. On the contrary in the HM loci, telomeres and subtelomeres there is not a significant enrichment in Sirt1 occupancy . The ability of SIRT1 to interact in different ways with different regions, despite the overexpression, may reflect in part its different ability to interact with yeast proteins.

In conclusion, we have hereby shown that *SIRT1* complementation in *sir2Δ* mutant cells exhibits heterogeneous profiles in molecular phenotypes, such as histone deacetylation mechanisms, that may trigger the repression effects on both RNA transcription and DNA recombination processes. These phenotypes do not involve all the expected silent loci, being HM loci and telomeres refractory to Sirt1 expression.

Studying the behavior of human Sirt1 in *S. cerevisiae* could be important to shed light on new aspects of this human protein. The study of protein partners of Sirt1 within the complemented loci, together with an evolutionary analysis on human homologs of yeast proteins, may bring to light in the future new protein partners of Sirt1 in humans. 

Finally this work demonstrates that human *SIRT1* gene is able to complement different molecular yeast phenotypes of the *sir2Δ* mutant with different efficiency. These observations indicate that there is a cross-talk between Sirt1 and yeast chromatin and that probably *in vivo* screenings, focused on H3K9Ac global decrease, would be possible in yeast. Since only *in vitro* assays are available for the screening of Sirt1 activators and inhibitors, *S.cerevisiae*, whose chromatin context is highly characterized, would be the first model for *in vivo* screening of molecules targeting this important protein.

## Materials and Methods

### Yeast strains, plasmids and oligo sequences

Yeast strains, plasmids and oligo sequences are listed in table S1.

### Culture media and conditions

Yeast cells were grown and manipulated according to standard protocols [47]. YPD medium (1% Bacto yeast extract, 2% Bacto peptone, 2% glucose) was used for auxotrophic strains lacking the *URA3* gene. For the maintenance of auxotrophic strains complemented with *URA3*-plasmids, we used minimal SD medium (0.67% Difco yeast nitrogen base without amino acids, 2% dextrose) with Dropout (DO) supplement lacking uridine. *SIRT1* induction experiments were carried out in minimal SD medium containing 2% glucose and then shifted in 2% galactose. For solid plates, 2% agar was added to SD medium. 

### RT-PCR

RNA from logarithmically growing cultures was isolated as previously described [47].A 1.5-μg amount of DNase I–treated RNA was subjected to cDNA synthesis, starting from 2.5 μM oligo(dT) for evaluation of *SIRT1, NTS1r, YFR057W, IRC7* and *HMLα1* mRNA expression levels (50ng/μl Random hexamers at 25°C 10min for *NTS2*), by incubation with 30 U of SuperScript III Reverse Transcriptase (Invitrogen, Cat.No. 18080-093) for 30 min at 50°C, followed by heating inactivation at 85°C for 5 min. 

The resulting cDNAs were amplified by PCR co-amplification using the following primer pairs: SIRT1-F/SIRT1-R, NTS2-F/NTS2-R each with ACT1-450-F/ACT1-450-R; NTS1r-F/NTS1r-R, YFR057W-F/ YFR057W-R, IRC7-F/IRC7-R, HML1α-F/ HML1α-R each co-amplified with ACT1-182-F/ACT1-182-R. 

PCR was performed under the following conditions: 95°C for 30 s, 60°C for 30 s, and 68°C for 1 min, with 18 cycles for *ACT1*, 24 cycles for *SIRT1*, *NTS1r* and *NTS2*, 27 cycles for *YFR057W*, *IRC7* and *HMLα1*. *NTS2* annealing was performed at 55°C. 

Taq polymerase (Eppendorf, Cat.No.2200320). [α-32P]dATP (Amersham, GE Healthcare, Cat.No.PB10204) was added to the reaction mixture (0.04 μCi/μl). Template titration for each sample was performed to evaluate the linear range of amplification. The amplified fragments were separated on a 6% polyacrylamide gel. For quantification ImageJ 1.43u was used. Each cDNA band intensity was normalized to *ACT1*. For mRNAs expression analysis data, average (with standard deviations, SD) refers to at least three independent biological replicas: (WT+; *sir2Δ−; sir2Δ+*)/(WT*−*). Student’s t test was applied for statistical analysis; α = 0.05. 

### ERC analysis

Yeast cells grown to exponential phase (OD600 0.5/ml) were lysed with lysis buffer (1% SDS, 100 mMNaCl, 10 mMTris pH 8.0, 1 mM EDTA) and glass beads (Sigma-Aldrich, G9268-500G) by vigorous shaking for 1 hour at 4°C. The DNA was then purified by three phenol–chloroform–isoamylic alcohol (24:24:1 [vol/vol/vol]) extractions followed by ethanol precipitation. Proteinase K (0.2 μg/μl) and RNase A (0.15 μg/μl) treatments were also performed. A 5-μg amount of the recovered DNA was run in 0.8% agarose gel electrophoresis (1.75 V/cm) and transferred onto a nylon membrane (GE Healthcare, Cat.No. RPN203B). Hybridization was performed with an rDNA probe annealing from −100 to −2400 base pairs upstream of the RNA polymerase I transcription start site. For image acquisition the Typhoon 9410 Variable Mode Imager (GE Healthcare) was used. The band intensities corresponding to ERCs were measured with ImageJ 1.42q (National Institutes of Health), and normalized to the hybridized bulk rDNA (loading control). ERC values were then divided by the calculated WT−level. Means and error bars refer to five independent biological replicates: (WT+; *sir2Δ−; sir2Δ+*)/(WT*−*). Student’s t test was applied for statistical analysis; α = 0.05 [40].

### Western blot

Yeast cells were grown to the exponential phase and lysed with NP40 buffer (0.2% NP40, 200 mM NaCl, 50 mM Tris pH 7.5, 1 mM PMSF, and protease inhibitors) and glass beads (Sigma-Aldrich, G9268-500G) by vigorous shaking for 1 h at 4°C. A 40-μg amount of protein extract was separated by a 13% SDS-polyacrylamide gel followed by immunoblotting. The PVDF membranes (Millipore, Cat.No.ISEQ20200) were incubated overnight at 4°C with primary antibodies: rat anti-Tubulin (Santa Cruz Biotechnology, sc-53030) was used at 1:5000 dilution; rabbit anti-acetyl H3K9, H4K12 at 1:7000 dilution (Upstate/Millipore, Cat.No.07-352 and 07-595); rabbit anti-acetyl H4K16 at 1:6000 (Santa Cruz Biotechnology, sc-8662-R); rabbit anti-acetyl H4Ct at 1:500 (Santa Cruz Biotechnology, sc-8658-R); rabbit anti-acetyl H3Ct at 1:500 (Santa Cruz Biotechnology, sc-10809); mouse anti-Flag 1:3000 (Sigma-Aldrich, Cat.No.F3165). Secondary antibodies: anti-rat IgG-HRP (Santa Cruz Biotechnology, sc-2006) was used at 1:10000 dilution, anti-rabbit IgG-HRP (Jackson ImmunoResearch, Cat.No.111-033-144) at 1:40000 dilution; Anti-Mouse IgG (whole molecule)-Peroxidase (Sigma-Aldrich, Cat.No.A9044) 1:20000. 

Detection was performed using SuperSignal West Pico Chemiluminescent Substrate (Thermo Scientific, Prod#34080). The integrated densities of each band were quantified with ImageJ 1.43u (National Institutes of Health). Densitometric analysis of relative histone acetylation levels were normalized to total histone levels (H3K9/H3Ct, H4K16/H4Ct, H4K12/H4Ct) and to WT- acetylation levels, WT−= 1 (Figure 2). Sirt1 Western Blot kinetics on *sir2Δ*(−,+,+*) strain (Figure S1) were normalized to alpha-Tubulin and to the 0h glucose-point before cells were shifted to galactose. Normalized values are reported as *sir2Δ*+/sir2Δ− ratio. Means and error bars refer to at least four independent biological replicas. Student’s t test was applied for statistical analysis; α = 0.05. 

### Chromatin immunoprecipitation

A 300-ml amount of culture was grown to exponential phase, crosslinked with 1% formaldehyde at room temperature for 15 min, and then incubated with 330 mM glycine for 10 min. Cells were then processed for ChIP as previously described [40]. A 350-μg amount of chromatin extract was incubated with 2.5 μl (2.5 μg) of antibodies against histone H3 or H4 C-terminal tail, anti–acetyl H4 Lys-16, anti–acetyl H3 Lys-9 (Millipore/Upstate, Cat.No.07-690, 04-858, 17-10101, 07-352), 5 μl (10 μg) of mouse anti-FLAG M1 (Sigma F3040) and mouse anti-IgG (as mock control, mouse a specific IgG Invitrogen 5292). Chromatin–antibody complexes were isolated with protein A–Sepharose beads (Amersham, GE Healthcare, Cat.No.17-0780-01) for 1.5 h at 4°C. The recovered DNA was resuspended in 200 μl for genomic sample (input) and in 50 μl for immunoprecipitated (IP) and beads only (BO) samples. Different amounts of DNA were used as template for PCR in order to obtain comparable autoradiographic signals (1 μl of a 1:20 dilution for input, and 1 μl for IP and BO). PCR was performed under the following conditions: 95°C for 30 s, 55°C for 30 s, and 68°C for 1 min, with 25 cycles for *ACT1*, TEL VI genes, *HMLalpha1* and 18 cycles for rDNA sequences. [α-32P]dATP was added to the reaction mixture (0.04 μCi/μl). For each immunoprecipitation three PCR reactions were done. The amplified fragments were separated on a 6% polyacrylamide gel. For quantification ImageJ 1.43u was used. Each set of experiments was repeated at least twice. Quantifications were performed as previously described [40].

Briefly, in Figure 5, acetylation fold enrichment values, for all regions, were calculated as follows: [rDNA(IP)/*ACT1*(IP)]/[rDNA(input)/ *ACT1*(input)]. In figure 5 the acetylation profiles were further corrected for the total amount of the histone H4 or H3. In this case the relative fold enrichment is defined as the ratio (acetylated histone)/(total histone) for values from the following calculation: [rDNA(IP)/*ACT1*(IP)]/[rDNA(input)/ *ACT1*(input)]. The isogenic *sir2Δ*- (with empty plasmid) strain value was then normalized to 1, obtaining the acetylation enrichment shown for the different mutants or conditions. Sirt1 relative fold enrichments in *sir2Δ+* strain (Figure 6) for all regions were calculated as: [locus(IP)/*ACT1*(IP)]/[locus(input)/ *ACT1*(input)]. Sirt1 profiles were reported as *sir2Δ+/sir2Δ*- with *sir2Δ-* =1 ; IP(*sir2Δ+*)/Input(*sir2Δ+*)]/[IP(*sir2Δ*−)/ input*sir2Δ*(−)]*.* The graphs show the mean and SD calculated from three technical replicates for at least three independent biological replicas.

### Spot-Test assay

Yeast cells were grown and collected at a density of 0.3-0.6 OD / ml. Cells, were then diluted to 4×103 cells/μl. Subsequent fivefold dilutions were made and 5 μl (8000 cells/μl) were spotted onto minimal medium plates containing glucose or galactose as carbon source, incubated at 30°C for 2–4 days and scanned. In the case of plates containing the alpha factor pheromone, this was used at 10 μg/ml final concentration [28].

## Supporting Information

Figure S1 ***SIRT1* Transcriptional and protein levels during galactose induction.** (A) RT-PCR to control the induced expression of *SIRT1* transcript in galactose (WT+, *sir2Δ*+, *sir2Δ*+ * versus WT− : *p < 5%). (B) Western blot kinetics in *sir2Δ* mutant with *SIRT1* construct (+), empty plasmid (−) and *SIRT1-H363Y* (+*) to check the presence of the protein during galactose induction. (C) Western blot quantification in *sir2Δ*- and in *sir2Δ*+ (Sirt1 levels: *sir*2Δ+ at hour 9 versus *sir2Δ*- or WT- in glucose at hour 0; **p < 1%).
(D) Construct for yeast expression with *SIRT1* or *SIRT1-H363Y* under the inducible promoter GAL1 in pYES2 background. (+: *SIRT1* construct; −: empty plasmid, +*: *SIRT1-H363Y*). (E) Yeast spot test analysis of growth phenotypes during plasmid repression and induction conditions (glucose and galactose, respectively). For WT-, *sir*2Δ-, *sir*2Δ+, *sir*2Δ+* strains five-fold serial dilutions were made and 5 μl were spotted onto minimal medium plates. Histograms (panels A and C) indicate averages and Std. Dev. bars from at least three independent biological replicates. Two−tailed t−test was applied for statistical analysis. Asterisks indicate statistically significant differences between analyzed strains. α = 0.05. (Percentages of p−value: *p< 5%, **p < 1%, ***p < 0.01%).
(TIF)

Table S1 **Strains, Plasmids and Oligonucleotides used in this work.**
(DOCX)
