# Correction: Functional Complementation of *sir2Δ* Yeast Mutation by the Human Orthologous Gene *SIRT1*

**DOI:** 10.1371/annotation/200805c7-c33f-415b-8f22-57d5e0455b5d

**Published:** 2014-01-09

**Authors:** Davide Gaglio, Anna D’Alfonso, Giorgio Camilloni

Figures 2-4 were incorrectly switched. The image currently appearing as Figure 2 belongs with the title and legend for Figure 3, the image currently appearing as Figure 3 belongs with the title and legend for Figure 4, and the image currently appearing as Figure 4 belongs with the title and legend for Figure 2.

