# Supplementary figures and images for: Functional Complementation of sir2Δ Yeast Mutation by the Human Orthologous Gene SIRT1
Source: PLoS One. 2013 Dec 11;8(12):e83114. doi: 10.1371/journal.pone.0083114 (PMC3859646; doi:10.1371/journal.pone.0083114)

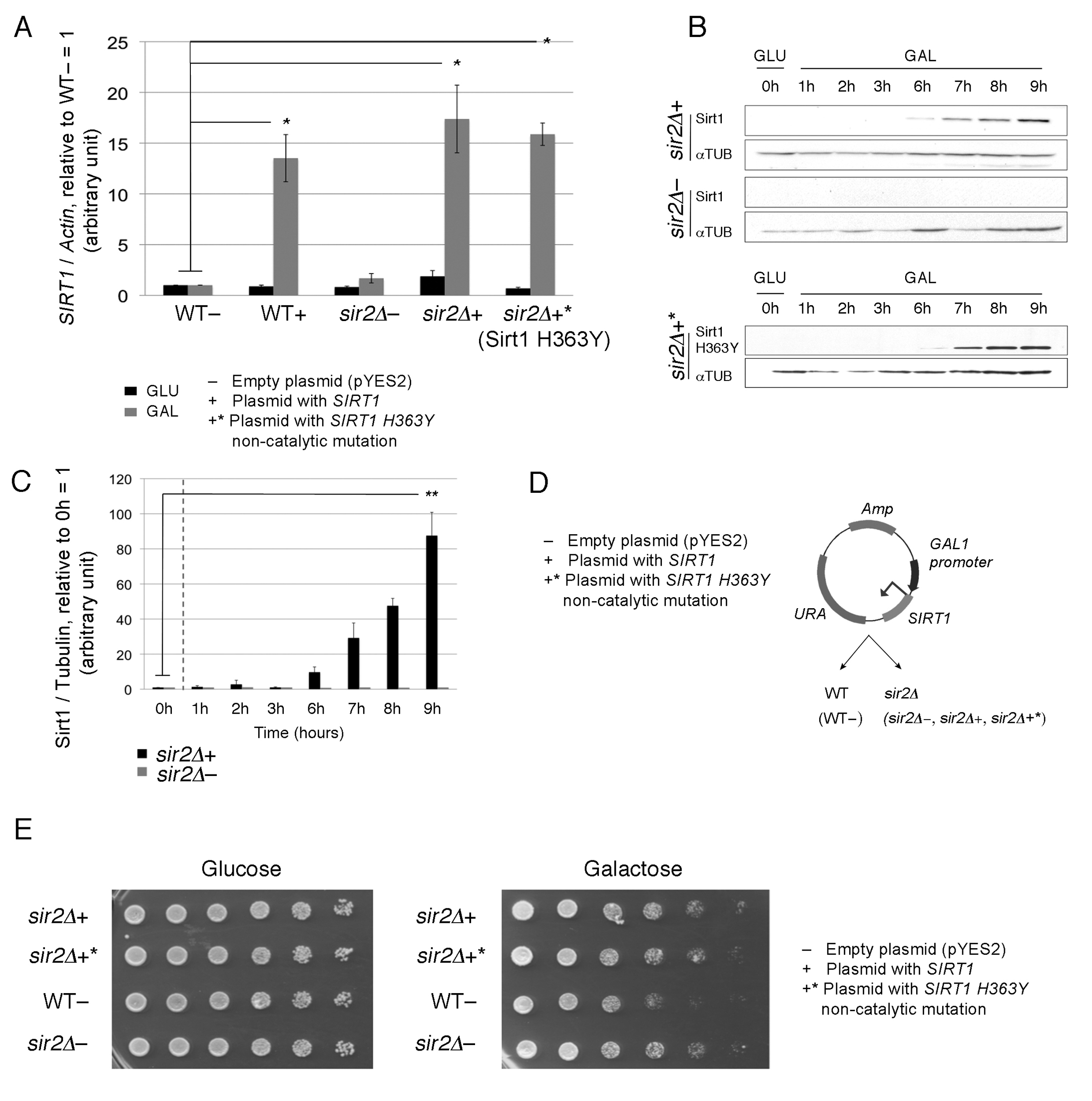

Supplement: Figure S1 — SIRT1 Transcriptional and protein levels during galactose induction. (A) RT-PCR to control the induced expression of SIRT1 transcript in galactose (WT+, sir2Δ+, sir2Δ+ * versus WT− : *p < 5%). (B) Western blot kinetics in sir2Δ mutant with SIRT1 construct (+), empty plasmid (−) and SIRT1-H363Y (+*) to check the presence of the protein during galactose induction. (C) Western blot quantification in sir2Δ- and in sir2Δ+ (Sirt1 levels: sir2Δ+ at hour 9 versus sir2Δ- or WT- in glucose at hour 0; **p < 1%). (D) Construct for yeast expression with SIRT1 or SIRT1-H363Y under the inducible promoter GAL1 in pYES2 background. (+: SIRT1 construct; −: empty plasmid, +*: SIRT1-H363Y). (E) Yeast spot test analysis of growth phenotypes during plasmid repression and induction conditions (glucose and galactose, respectively). For WT-, sir2Δ-, sir2Δ+, sir2Δ+* strains five-fold serial dilutions were made and 5 μl were spotted onto minimal medium plates. Histograms (panels A and C) indicate averages and Std. Dev. bars from at least three independent biological replicates. Two−tailed t−test was applied for statistical analysis. Asterisks indicate statistically significant differences between analyzed strains. α = 0.05. (Percentages of p−value: *p< 5%, **p < 1%, ***p < 0.01%). (TIF) [file pone.0083114.s001.tif]
