# Supplementary material for: Functional Complementation of sir2Δ Yeast Mutation by the Human Orthologous Gene SIRT1
Source: PLoS One. 2013 Dec 11;8(12):e83114. doi: 10.1371/journal.pone.0083114 (PMC3859646; doi:10.1371/journal.pone.0083114)
Supplement: Table S1 — Strains, Plasmids and Oligonucleotides used in this work. (DOCX) [file pone.0083114.s002.docx]

**Table S1**

**Strains**

| *Strains* | *Genotype* | *Background (source)* | |
| --- | --- | --- | --- |
| **WT** | Mat a; ade2-1; ura3-11; trp1-1; leu2-3.112; Can1-100 | W303-1a | |
| **WT −** | Mat a; ade2-1; ura3-11; trp1-1; leu2-3.112; Can1-100; pYES2 | WT strain (W303-1a) with pYES2 Plasmid. | |
| **WT +** | Mat a; ade2-1; ura3-11; trp1-1; leu2-3.112; Can1-100; pDGS1 | WT (W303-1a) strain with pDGS1 Plasmid. | |
| ***sir2Δ*** | Mat a; ade2-1; ura3-11; trp1-1; leu2-3.112; Can1-100; *sir2*::LEU2 | W303-1a (NOY1045, kindly  provided by M. Nomura). | |
| ***sir2Δ* −** | Mat a; ade2-1; ura3-11; trp1-1; leu2-3.112; Can1-100; sir2::LEU2; pYES2 | *sir2Δ* (W303-1a) strain with pYES2 plasmid. | |
| ***sir2Δ* +** | Mat a; ade2-1; ura3-11; trp1-1; leu2-3.112; Can1-100; sir2::LEU2; pDGS1 | *sir2Δ* (W303-1a) strain with pDGS1 Plasmid. | |
| ***sir2Δ* +*** | Mat a; ade2-1; ura3-11; trp1-1; leu2-3.112; Can1-100; sir2::LEU2; pDGS1-H363Y | | *sir2Δ* (W303-1a) strain with pYES2 plasmid with pDGS1-H363Y Plasmid. |

**−** = empty plasmid pYES2

**+** = pYES2 backbone with FLAG-*SIRT1* insert (called pDGS1)

+* = pYES2 backbone with FLAG-*SIRT1-H363Y* insert (called pDGS1-H363Y)

**Plasmids**

| *Plasmid* | *Features* | *Reference* |
| --- | --- | --- |
| **Addgene plasmid 1791** | pECE mammalian  expression vector; *SIRT1*-FLAG | Brunet et al.; Science, 2004. |
| **pYES2** | 2µ, URA3, AMP, GAL1 promoter, Poli-Linker CYC1 terminator. | T.Rinaldi et al.; Mol Biol Cell. 1998. |
| **pDGS1** | 2µ, URA3, AMP, GAL1 promoter, Poli-Linker, CYC1 terminator, h*SIRT1*-FLAG. | This study. |
| **pYMG1** | Centromeric CEN6/ARS4.  pRS316 backbone + 2212bp yeast rDNA fragment. | F.Cioci et al.; J. Mol. Biol. (2002). |
| **pDGS1-H363Y** | 2µ, URA3, AMP, GAL1 promoter, Poli-Linker, CYC1 terminator, hSIRT1-H363Y-FLAG. | This study. |
| **Addgene plasmid 1791 SIRT1-H363Y** | pECE mammalian  expression vector; SIRT1-H363Y-FLAG | Sirt1-H363Y,deacetylase domain mutation.  Brunet et al.; Science, 2004. |

Cioci F, Vogelauer M, Camilloni G. Acetylation and accessibility of rDNA chromatin in Saccharomyces cerevisiae in (Delta)top1 and (Delta)sir2 mutants. J Mol Biol 2002;322(1):41-52.

Rinaldi T, Ricci C, Porro D, Bolotin-Fukuhara M, Frontali L. A mutation in a novel yeast proteasomal gene, RPN11/MPR1, produces a cell cycle arrest,overreplication of nuclear and mitochondrial DNA, and an altered mitochondrial morphology. Mol Biol Cell 1998; 9(10):2917-31.  **Oligonucleotides**

*Oligonucleotide Oligonucleotide sequence (5′ to 3′ direction)*

**ACT1-182**-**F**  ACGTTCCAGCCTTCTACGTTTCCA

**ACT1-182-R** AGTCAGTCAAATCTCTACCGGCCA

**ACT1-450-F** GGTATTGTCACCAACTGGGACGAT

**ACT1-450-R** GAAGTCCAAGGCGACGTAACATAG

**SIRT1-F** GACAACTTGTACGACGAAGACGAC

**SIRT1-R** GGAGTCCAGTCACTAGAGCTTGCA

**YFR057W-F** CTATAGTAAGTGCTCGGCCAAGTC

**YFR057W-R** CTCTTCTGAGACGAAGTCGTTGCT

**NTS1r-F** GCACCATCAGAGCGGCAAAC

**NTS1r-R** CGCTGCCTCTCTGGAAC

**HML1α-F** CTTCCCAATATCCGTCACCACGTA

**HML1α-R** TCCAGATTCCTGTTCCTTCCTCTC

**IRC7-F** CAACCGTCATTTTCCTCGAAAGCC

**IRC7-R** GCAATGCTAATTGACAGTCCTCGG

**EPRO-F** TGTTAGTGCAGGAAAGCGGGAAGGA

**EPRO-R** GCACTATCCAGCTGCACTCTTCTTC

**CPRO-F** AATAGTGAGGAACTGGGTTACCCG

**CPRO-R** TTGTACTCCATGACTAAACCCCCC

**NTS2-F** ATGTTCAGTAGGTGGGAGTGAGAG

**NTS2-R** CATCCGGTGCCGTAAATGCAAAAC

***SIRT1* molecular sub-cloning**

Given the presence of a HindIII restriction site at the N-terminal of FLAG-h*SIRT1 (or FLAG-hSIRT1-H363Y)* in addition to that inside the gene, we performed a controlled kinetics restriction in order to isolate the complete insert from the plasmid addgene 1791. First, 1791 was digested with XbaI: 390 ng/μl plasmid, cut Buffer H (0.05 Tris-HCl M, 0.1 M NaCl, 0.01 M MgCl2, 1mM [dithiothreitol](http://www.google.it/search?hl=it&biw=1053&bih=610&sa=X&ei=AE1oUNejDY_54QStvICYAw&ved=0CB0QvwUoAQ&q=dithiothreitol&spell=1) pH 7.5 at 37°C), 1 U/μl XbaI (Roche) at 37°C for 3h. Then the restricted plasmid was digested with HindIII: 390 ng/μl plasmide, cut Buffer B (10 Tris-HCl mM, 0.1 M NaCl, 5 mM MgCl2, 1 mM 2-mercaptoethanol pH 8 at 37°C), 0.2 U/μl di HindIII (Roche) performer at 37°C. We performed three samples (0.5h, 1h, 1.5h) to check the kinetics point where M2-FLAG-h*SIRT1* was not cut inside the coding sequence. The pYES2 plasmid was digested as 1791 palsmid, but the HindIII reaction was performed, without sampling during the kinetics.

The pYES2 plasmid backbone was subjected to dephosporilation reaction as follows: 80 ng/μl of restricted pYES2, dephosphorilation buffer 1X (0.05 M Tris-HCl, 0.1 mM EDTA, pH8.5 at 20°C), 0.06 U/μl of alkaline phosphatase (Roche), at 37°C for 30 minutes. DNA ligase reaction between dephosphorilated pYES2 backbone and FLAG-h*SIRT1* insert, both HindIII-XbaI digested, was carried out in these conditions: 2 ng/μl pYES2 plasmid, 2 ng/μl FLAG-h*SIRT1* insert, Buffer T4 DNA ligase (50 mM Tris-HCl, 10 mM MgCl2, 10 mM DTT, 1 mM ATP), 0.1 U/μl T4 DNA ligase. Ligase reaction was performed at room temperature for 4 hours. Cell transformation was performed with 5 μl of ligase reaction and 50 μl of HIT-DH5α competent cells from Invitrogen. Cells were vortexed, placed in ice for 20 minutes and then at 42°C for 1 minute. Positive clones were selected on LB plates selective for ampiciliin resistance (1% Bacto peptone, 0.5% yeast extract, 0.5% NaCl, 2% Agar, 0.05 mg/ml ampicillin). Cells were grown at 37°C for 15 hours. Clones were controlled by enzymatic restriction and PCR for their correct integration in the backbone plasmid.
